# The Effect of Sleep Disruption on Cardiometabolic Health

**DOI:** 10.3390/life15010060

**Published:** 2025-01-07

**Authors:** SeokHyun Hong, Da-Been Lee, Dae-Wui Yoon, Seung-Lim Yoo, Jinkwan Kim

**Affiliations:** 1 Sleep Medicine Institute, Jungwon University, Goesan-gun 28204, Chungcheongbuk-do, Republic of Korea; mlmljk3620@gmail.com (S.H.); dabin050912@korea.ac.kr (D.-B.L.); ysl990628@naver.com (S.-L.Y.); 2 Department of Biomedical Laboratory Science, College of Health Science, Jungwon University, Goesan-gun 28204, Chungcheongbuk-do, Republic of Korea

**Keywords:** sleep disruption, metabolic dysfunction, cardiovascular disease, inflammation

## Abstract

Sleep disruption has emerged as a significant public health concern with profound implications for metabolic health. This review synthesizes current evidence demonstrating the intricate relationships between sleep disturbances and cardiometabolic dysfunction. Epidemiological studies have consistently demonstrated that insufficient sleep duration (<7 h) and poor sleep quality are associated with increased risks of obesity, type 2 diabetes, and cardiovascular disease. The underlying mechanisms are multifaceted, involving the disruption of circadian clock genes, alterations in glucose and lipid metabolism, the activation of inflammatory pathways, and the modulation of the gut microbiome. Sleep loss affects key metabolic regulators, including AMPK signaling and disrupts the secretion of metabolic hormones such as leptin and ghrelin. The latest evidence points to the role of sleep-induced changes in the composition and function of gut microbiota, which may contribute to metabolic dysfunction through modifications in the intestinal barrier and inflammatory responses. The NLRP3 inflammasome and NF-κB signaling pathways have been identified as crucial mediators linking sleep disruption to metabolic inflammation. An understanding of these mechanisms has significant implications for public health and clinical practice, suggesting that improving sleep quality could be an effective strategy for preventing and treating cardiometabolic disorders in modern society.

## 1. Introduction

Sleep is a fundamental biological process that plays a crucial role in maintaining physical, mental, and cognitive health in the human body. However, a significant proportion of the population experiences insufficient sleep due to shortened duration and fragmented, low-quality sleep. A growing body of evidence has demonstrated that approximately 30–40% of adults do not obtain the recommended 7 or more hours of nightly sleep [1,2]. This concerning trend has coincided with a marked increase in the prevalence of obesity and obesity-related metabolic diseases, prompting investigations into the potential role of sleep disruption in the development of metabolic dysfunction [3,4,5]. Two key factors regulating sleep timing and functioning, circadian biology and sleep homeostasis, exert a profound influence on physiological processes over a 24-h period. Core clock genes not only orchestrate the rhythmic expression of rate-limiting proteins and enzymes involved in substrate utilization and storage across tissues [6,7,8], but they also regulate metabolism and energy homeostasis in peripheral tissues [7]. Furthermore, sleep modulates the secretion of appetite hormones, such as leptin and ghrelin, that affect hunger sensations and feeding patterns [5,9]. A substantial body of evidence from both experimental and epidemiological studies suggests that sleep disruption is associated with adverse metabolic effects, including reduced glucose tolerance, insulin sensitivity, and elevated markers of inflammation. These changes may contribute to an increased risk of developing diabetes and obesity [4,5].

Bidirectional relationships further complicate examining causal pathways in observational data. It is well established that obesity is an independent risk factor for the development of sleep disorder breathing (SDB), while weight loss significantly improves symptoms [5,10]. Diabetics frequently report sleep complaints and experience circadian alterations that may exacerbate metabolic disturbances [4,9]. The disentanglement of this two-way association between poor metabolic functioning and sleep disruption remains an active area of investigation. Moreover, emerging research suggests that common risk loci related to circadian rhythms may be associated with metabolic traits underlying obesity, diabetes, and cardiovascular disorders through genome-wide association studies [11,12]. Integrating insights across biochemical, physiological, genetic, and epidemiological studies are likely to yield novel targets for preventive and therapeutic interventions aimed at improving sleep as a modifiable lifestyle factor that mitigates obesity and cardiometabolic diseases. Thus, the objective of this comprehensive review is to address several key research objectives: Firstly, this review examines the bidirectional relationship between sleep disruption and cardiometabolic dysfunction, with a specific focus on the impact of insufficient sleep duration on obesity risk, the association between sleep disruption and type 2 diabetes development, and the relationship between sleep disturbances and cardiovascular morbidity and mortality. Secondly, the objective is to elucidate the molecular mechanisms underlying the effect of sleep disruption on metabolism. This entails examining the role of circadian clock genes in metabolic regulation, the impact of sleep disruption on glucose and lipid metabolism, the relationship between sleep disruption and inflammatory pathways, and the interaction between sleep disruption and the gut microbiome.

## 2. Short Sleep Duration and the Increased Risk of Obesity

The National Sleep Foundation (NSF) guidelines have established guidelines recommending a sleep duration of 7–9 h per night for adults aged 18–64 years. The guidelines also state that sleep duration of 6 h or less and 10 h or more may have a detrimental impact on health and well-being [13]. Nevertheless, it is estimated that approximately one in four individuals globally experience some form of sleep-related issue [2]. A growing body of epidemiological studies has demonstrated a relationship between reduced sleep duration and an increased risk of obesity and weight gain over time [1] (Table 1). Cross-sectional studies have shown varying results regarding the relationship between sleep duration and obesity, ranging from linear to U-shaped associations. The inconsistency in findings can be attributed to multiple factors including differences in methods such as sleep assessment, obesity measurement, and adjustment for confounding factors. However, multiple systematic reviews and meta-analyses of cross-sectional and prospective cohort studies provide consistent evidence that short sleep (often defined as <7 h per night) is associated with elevated body mass index (BMI), higher odds of obesity or being overweight, and greater long-term weight gain compared to normal sleep duration [1,4,5]. The Nurses’ Health Study (NHS), a landmark investigation following 68,183 women for 16 years, revealed that participants who slept ≤5 h per night had a 15% higher risk of becoming obese compared to those who slept 7 h [14]. The results showed that women who slept for 5 h or less gained 1.14 kg more over the follow-up period compared to those who slept for 7 h [14]. Moreover, each hour reduction in sleep was linked to an average increase of 0.35 kg in weight gain at the end of the follow-up period. These findings demonstrate a significant, dose-dependent relationship between restricted sleep and weight gain over time. The Whitehall Ⅱ prospective cohort study, which followed 5021 participants for a period of 5 years, revealed significant associations between sleep duration and the risk of obesity in a cross-sectional study after adjustment for confounding factors. Nevertheless, in prospective analysis, a short duration of sleep was not notable alterations in BMI or waist circumference, nor was it linked to the incidence of obesity. More recently, prospective cohort studies have consistently demonstrated a significant association between short sleep duration and an increased risk of obesity. These studies offer stronger evidence than cross-sectional studies due to their temporal design and ability to establish causality. A recent meta-analysis by Bacaro et al. examined 12 studies comprising a total of 154,936 participants, with a specific focus on adult populations [15]. The analysis confirmed that a short sleep duration of ≤6.5 h was significantly associated with an increased risk of future obesity (OR = 1.41, 95% CI: 1.18–1.69), whereas a longer sleep duration was not associated with this risk [15]. The most comprehensive analysis, conducted by Deng et al., included 29 studies with 57,848 children and adolescents. That analysis demonstrated that that short sleep duration was a significantly increased risk factor for obesity (RR = 1.57, 95% CI: 1.36–1.81) [16].

## 3. Sleep Disruption and Increased Risk of Type 2 Diabetes

A growing body of epidemiologic and experimental evidence has linked disrupted sleep patterns to an increased risk of type 2 diabetes (T2DM) (Table 2) [17,18,19]. Specifically, insufficient sleep duration, poor sleep quality, and sleep disturbances have been linked to metabolic dysfunction and the development of diabetic phenotypes. Several large prospective cohort studies have demonstrated an association between short sleep duration and an elevated incidence of diabetes over the course of multiple years of follow-up [4,5,10]. For instance, a study of the Nurses’ Health Study of over 70,000 female nurses revealed that those who reported sleeping less than 5 h had a 35% increased risk of developing diabetes over a 10-year period compared to nurses who slept 7 h, after controlling for confounding factors [20]. A similar result was observed in the National Health Interview Survey of U.S adults, which included 130,943 participants [21]. A meta-analysis of data from multiple perspective studies showed a U-shaped relationship between sleep duration and the risk of developing T2DM. The lowest risk of developing T2DM was observed in individuals who reported an average sleep duration of 7–8 h per day [22]. Both short and long sleep durations were significantly associated with an increased risk of T2DM, highlighting the importance of appropriate sleep duration in delaying or preventing T2DM. A dose–response relationship was observed, with the odds of developing diabetes increasing progressively below the optimal range of 7 h. It is plausible that a lack of sleep may contribute to an array of physiological processes that could potentially lead to an increased risk of developing glucose intolerance, insulin resistance, and a reduction in pancreatic β-cell function [5,23,24]. Additionally, elevated levels of inflammatory markers and weight gain have also been linked to insufficient sleep [5,25,26]. Qualitative aspects of sleep disruption, in addition to short duration, are also associated with an increased risk of diabetes. The modulation of metabolic outcomes may be influenced by various sleep parameters, including sleep latency, efficiency, timing, regularity, and continuity. The extant evidence indicates that sleep quality is associated with higher hemoglobin A1c levels, which are indicative of poor glycemic control [27,28]. Specifically, studies have demonstrated that a significant relationship exists between elevated hemoglobin A1c levels, sleep quality, and sleep duration in patients with prediabetes [28]. Furthermore, insomnia symptoms, such as difficulty maintaining sleep, have been associated with elevated HbA1c levels in Japanese men [27]. A meta-analysis study showed that individuals with type 1 diabetes who experienced disrupted sleep patterns tended to exhibit poorer glycemic control and lower HbA1c levels [29].

Sleep disordered breathing (SDB) is a condition that is defined by the presence of abnormal respiratory patterns during sleep. It has been identified as a significant risk factor for metabolic dysfunction, including obesity and T2DM [10,24]. Obstructive sleep apnea (OSA), the most prevalent form of SDB, is marked by repeated episodes of partial or complete upper airway obstruction leading to intermittent hypoxia, sleep fragmentation, and sympathetic activation [30]. Over the past two decades, numerous studies have consistently demonstrated an association between OSA and a higher risk of diabetes across diverse populations and age groups [5,10]. One of the earliest and most influential prospective studies to investigate the link between OSA and the incidence of diabetes was the Wisconsin Sleep Cohort Study [31]. The study cohort comprised 1387 participants who were initially free of diabetes at the baseline assessment between 1988 and 1994. Polysomnography was conducted to evaluate the severity of OSA based on the apnea-hypopnea index (AHI). In the cross-sectional study, after adjusting for age, sex, and body habitus, the odds ratio for having a physician diagnosis of diabetes was 2.30 (95% confidence interval, 1.28–4.11; *p* = 0.005) with an AHI of 15 or more compared to an AHI of less than 5. However, the study did not establish whether SDB was a causal factor in the development of diabetes in a prospective setting. The Nurses’ Health Study, a large prospective cohort of 69,852 female nurses aged 40–65 years, investigated the association between self-reported symptoms of SDB and the subsequent risk of physician-diagnosed diabetes over a 10-year follow-up period [32]. After adjusting for other factors that contribute to the risk of developing diabetes and for variables related to sleep, the researchers found that women who reported regular snoring at baseline had a 2.25-fold increased relative risk of developing diabetes compared to non-snorers. In order to investigate the association between OSA and incident diabetes, Kendzerska et al. also conducted a study using a large clinical cohort and comprehensive health administrative data [33]. The study included 8678 adults without diabetes who underwent a diagnostic sleep study in Toronto, Canada between 1994 and 2010. The severity of OSA was evaluated by means of the AHI and other physiological parameters. The occurrence of incident diabetes was identified through the application of validated algorithms to health administrative data. During a median follow-up of 67 months, 1017 (11.7%) participants had developed diabetes. In fully adjusted models, severe OSA (AHI > 30) was associated with a 30% elevated risk of developing diabetes compared to those without OSA (AHI < 5). The association between the AHI and diabetes risk was attenuated by an increasing age and BMI. The findings of that study provided compelling evidence of a distinct correlation between the severity of OSA and incident T2DM. This study addressed the limitations of previous research by utilizing a large sample size, a long follow-up period, and a comprehensive evaluation of OSA parameters. These findings underscored the importance of considering the OSA severity and related physiological parameters when assessing the risk of developing diabetes. In a recent population-based study of middle-aged and older adults conducted by Siddiquee A et al. from the Korean Cohort Study, an association between OSA severity and the risk of incidence of T2DM was identified. The study revealed that individuals with moderate–severe OSA exhibited a 1.5-fold increased risk of developing T2DM over an 8-year period [34]. The relationship between sleep disruption, particularly OSA, and the risk of T2DM has been investigated in several meta-analyses of prospective cohort studies. In light of the mounting evidence from individual cohort studies, Wang et al. conducted one of the most comprehensive meta-analyses on this topic, encompassing six prospective cohort studies with a total of 5953 participants [35]. The study found that individuals with moderate to severe OSA exhibited a markedly increased risk of developing T2DM (RR = 1.63, 95% CI: 1.09–2.45) in comparison to those without OSA. The association between OSA and an elevated risk of developing diabetes remained statistically significant even after adjusting for potential confounders such as age, sex, and BMI. A more recent meta-analysis conducted by Qie et al. synthesized data from 16 prospective cohort studies comprising a total of 338,912 participants [36]. The analysis demonstrated a linear relationship between OSA and the risk of T2DM. Specifically, the pooled relative risk of T2DM was 1.40 (95% CI, 1.32–1.48) for OSA in the binary meta-analysis and 1.08 (1.01–1.14) for each five-event/h increase in the apnea-hypopnea index (AHI) value. The findings of that study indicated a positive linear association between OSA and the risk of developing T2DM.

**Table 2:** The relationship between sleep disruption and the risk of developing T2DM in population-based studies.

Study	Population	Study Design	Follow-Up Period	Sleep Parameter Measured	Primary Outcomes	Risk Ratio (95% CI)	Reference
Nurses’ Health Study	70,000 female nurses; US	Prospective cohort	10 years	Self-reported sleep duration	T2DM incidence	1.35 for ≤5 h sleep	[20]
National Health Interview Survey	130,943 adults; Mixed ethnicity	Population survey	Various	Sleep duration and quality	Diabetes risk	Significant linear association	[21]
Korean Cohort	Middle-aged adults; Asian population	Prospective cohort	8 years	OSA severity (AHI)	T2DM development	1.5 for moderate–severe OSA	[34]
Wang et al.	5953 participants; multiple ethnicities	Meta- analysis	Various	OSA presence and severity	Diabetes risk	1.63 (1.09–2.45)	[35]
Qie et al.	338,912 subjects; global population	Meta- analysis	Various	OSA severity levels	T2DM risk	1.40 (1.32–1.48)	[36]

## 4. Sleep Disruptions and Cardiovascular Morbidity and Mortality

Sleep is a crucial aspect of overall health and well-being. Sleep complaints, including insomnia, short sleep duration, and poor sleep quality, have been increasingly recognized as significant risk factors for cardiovascular diseases (CVDs) and mortality. CVDs, including coronary heart disease, stroke, and heart failure, are the leading cause of mortality worldwide [37]. The identification of modifiable risk factors for CVDs is of paramount importance for the development of effective prevention and treatment strategies. Epidemiological studies have played an instrumental role in establishing the association between sleep complaints and cardiovascular outcomes, providing valuable insights into the potential predictive value of sleep complaints in assessing cardiovascular risk (Table 3).

### 4.1. Insomnia and Cardiovascular Disease

Insomnia is a prevalent sleep disorder that impairs the ability to initiate or maintain sleep, often accompanied by daytime impairments such as fatigue, mood disturbances, and cognitive dysfunction. The relationship between insomnia and cardiovascular mortality has been the subject of investigation in several epidemiological studies. A meta-analysis conducted by Sofi and colleagues included 13 prospective cohort studies comprising a total of 122,501 participants [38]. The study revealed that individuals with insomnia exhibited a 45% elevated risk of cardiovascular mortality compared to those without insomnia (HR: 1.45, 95% CI: 1.29–1.62). The association remained statistically significant after adjusting for potential confounding factors, including age, sex, and comorbidities, indicating an independent relationship between insomnia and cardiovascular mortality. A prospective study conducted by Laugsand et al. with 52,610 participants from the Norwegian HUNT study lent support to the association between insomnia and the risk of acute myocardial infarction (AMI) [39]. The study employed a three-item questionnaire to address insomnia symptoms. The study found that individuals who encountered difficulties initiating sleep on a nearly nightly basis exhibited a hazard ratio of 1.45 (95% CI: 1.18–1.80) for AMI, after adjusting for a multitude of confounding factors. Individuals who experienced difficulties maintaining sleep almost every night had a hazard ratio of 1.30 (95% CI: 1.01–1.68), and those who experienced a feeling of nonrestorative sleep more than once a week had a hazard ratio of 1.27 (95% CI: 1.03–1.57) compared to those who never experienced these sleep difficulties. The number of insomnia symptoms was dose-dependently associated with the risk of AMI, indicating the potential role of insomnia as an early predictor of cardiovascular risk. The precise mechanism by which insomnia may contribute to cardiovascular mortality remains unclear. However, several potential pathways have been proposed. Insomnia has been linked to increased sympathetic nervous system activity, which can lead to elevated blood pressure, heart rate, and endothelial dysfunction [40]. Additionally, insomnia has been associated with increased levels of pro-inflammatory cytokines, including interleukin-6 and C-reactive protein, which are established risk factors for CVDs [41,42]. Moreover, insomnia may contribute to the development of other cardiovascular risk factors, such as obesity, diabetes, and dyslipidemia, through its impact on neuroendocrine and metabolic function [42].

### 4.2. Short Sleep Duration and Cardiovascular Disease

Short sleep duration, typically defined as sleeping less than 6 h per night, has been consistently associated with an increased risk of cardiovascular mortality in epidemiological studies. A large cohort study by Cappuccio et al. [43] pooled data from 15 prospective studies with a total of 474,684 participants and found that individuals who slept less than 6 h per night had a 48% higher risk of developing or dying of CHD compared to those who slept 6 to 8 h (HR: 1.48, 95% CI: 1.22–1.80). The observed association was consistent across different geographical regions and age groups, which suggests that there is a robust relationship between short sleep duration and cardiovascular mortality. A more recent meta-analysis conducted by Itani et al. included 153 prospective cohort studies with a total of 5,172,710 participants [1]. The study found that a short sleep duration, defined as less than 6 h, was associated with an elevated risk of mortality (RR: 1.12, 95% CI: 1.08–1.16), as well as an increased risk of CVD (RR: 1.16, 95% CI: 1.10–1.23) and coronary heart disease (RR: 1.26, 95% CI: 1.15–1.38) when compared to the reference sleep duration of 7 to 8 h. The study also found a U-shaped association between sleep duration and cardiovascular mortality. The evidence suggests that both short and long sleep durations are associated with increased cardiovascular mortality, highlighting the significance of maintaining an optimal appropriate amount of sleep to avoid negative impacts on cardiovascular health. The underlying mechanisms linking short sleep duration to cardiovascular mortality are complex and involve multiple interconnected pathways, including the activation of the sympathetic nervous system, metabolic dysregulation, endothelial dysfunction, and increased inflammation [1,43,44]. These pathways can work independently or in combination to increase the risk of cardiovascular incidents and mortality in individuals with short sleep duration. One of the key mechanisms through which short sleep duration contributes to cardiovascular mortality is the activation of the sympathetic nervous system. It has been demonstrated that sleep deprivation leads to an increase in sympathetic nervous system activity, which in turn results in elevated blood pressure, an increased heart rate, and enhanced platelet aggregation [44]. The chronic activation of the sympathetic nervous system has been demonstrated to contribute to the development of hypertension, a significant risk factor for CVD. Elevated blood pressure over time can result in damage to the blood vessels, an increased workload on the heart, and acceleration of atherosclerosis. Such effects serve to increase the risk of cardiovascular incidents and mortality [45]. In addition to its effects on the sympathetic nervous system, short sleep duration has been linked to metabolic dysregulation. Insufficient sleep has been associated with impaired glucose tolerance, insulin resistance, and alterations in appetite-regulating hormones, including leptin and ghrelin [5,17,22]. Such metabolic alterations may contribute to the development of obesity and type 2 diabetes, both of which are well-established risk factors for CVD. Another potential mechanism linking short sleep duration to cardiovascular mortality is endothelial dysfunction [46,47]. The endothelium is a thin layer of cells that lines the interior of blood vessels and plays a crucial role in maintaining vascular health by regulating vasodilation, inflammation, and thrombosis [45]. Endothelial dysfunction is characterized by a reduction in the capacity of the endothelium to facilitate vasodilation and an increased propensity for inflammatory process and thrombosis. This can contribute to the pathogenesis of CVD [45,46].

### 4.3. Poor Sleep Quality and Cardiovascular Disease

In addition to insomnia and short sleep duration, poor sleep quality has also been identified as a predictor of cardiovascular mortality in epidemiological studies [48]. Poor sleep quality can be assessed by subjective measures, such as self-reported sleep satisfaction or sleep disturbances, or by objective parameters, such as sleep efficiency (the percentage of time spent asleep relative to the total time in bed) and sleep fragmentation (the number of awakenings or arousals during sleep). A prospective study by Bertisch et al. [49] included 4994 participants from the Sleep Heart Health Study (median follow-up period of 11.4 years), who completed questionnaires and home polysomnography (PSG) between 1994 and 1998. The incidence of CVD was found to be higher in the incident CVD group compared with the reference group (HR: 1.29, 95% CI: 1.00, 1.66). However, neither the insomnia or poor sleep only group nor the short sleep only group demonstrated an association with higher incident CVD after adjusting for potential confounders. The study underscores the value of including objective measures of sleep quality in addition to subjective complaints when assessing cardiovascular risk. In a separate study, Hu W et al. conducted a prospective analysis of the UK biobank data set to examine whether lifestyle, psychosocial, and biological factors mediated the association. The study demonstrated that poor sleep quality, as assessed by a sleep score incorporating five sleep behaviors, was associated with an increased risk of both all-cause mortality (HR: 1.098, 95% CI: 1.05, 1.14) and CVD mortality (HR: 1.13, 95% CI: 1.04, 1.24), suggesting that interventions aimed at promoting healthy lifestyle and psychosocial health may help to reduce the risk of CVD [50]. A recent study involving 4058 participants in the Sleep Heart Health Study and 2193 participants in the MrOS (Osteoporotic Fractures in Men Study) Sleep Study produced interesting results. The study found that lower delta wave entropy during sleep was associated with a higher risk of CVD mortality (SHHS: HR: 1.94; 95% CI: 1.18, 3.18; MrOS: HR: 1.66; 95% CI: 1.12, 2.47) after adjustment for covariates [51]. Most importantly, the findings of that study suggest that the disruption of delta wave activity during sleep may serve as a useful metric for identifying individuals at an increased risk of CVD mortality. The potential mechanisms linking poor sleep quality to CVD mortality are likely multifactorial and may overlap with those of insomnia and short sleep duration. Poor sleep quality has been associated with increased sympathetic nervous system activity, endothelial dysfunction, and inflammation [1,43,44]. Additionally, poor sleep quality can lead to daytime sleepiness and fatigue, which can negatively affect lifestyle behaviors such as physical activity and diet, further contributing to cardiovascular risk [42]. In summary, epidemiological studies have consistently demonstrated that sleep complaints, including insomnia, short sleep duration, and poor sleep quality, are associated with an increased risk of CVD mortality. These findings underscore the importance of considering sleep as a critical factor in cardiovascular health and the need to integrate sleep assessment and management into cardiovascular risk prediction and prevention strategies.

**Table 3:** The relationship between sleep disruption and cardiovascular morbidity and mortality.

Sleep Parameter	Study Design	Sample Characteristics	Duration	Outcomes	Risk Ratio/HR (95% CI)	Reference
Insomnia	Meta-analysis of 13 studies	122,501 adults; multiple countries	Various	CV mortality	1.45 (1.29–1.62)	[38]
Insomnia symptoms	Prospective study	52,610 adults	11.4 years	Acute MI	1.45 (1.18–1.80)	[39]
Short sleep (<6 h)	Meta-analysis	474,684 adults	Various	CHD risk	1.48 (1.22–1.80)	[43]
Poor sleep quality	UK Biobank	205,654 adults	13 years	CVD mortality	1.13 (1.04–1.24)	[50]
Delta wave disruption	SHHS/MrOS combined analysis	6251 participants	Extended follow-up	CVD mortality	SHHS: 1.94 (1.18–3.18); MrOS: 1.66 (1.12–2.47)	[51]

## 5. Molecular Mechanism Underlying Sleep Disruption and Metabolic Dysfunction

### 5.1. Circadian Clock System and Metabolic Regulation

The circadian clock system is an endogenous temporal mechanism that is integral to the modulation of a variety of physiological processes, of which metabolism is a prominent example (Table 4). This system allows organisms to anticipate and adapt to daily environmental changes and to optimize their metabolic functions accordingly [11,52]. The circadian clock system is comprised of a primary clock located in the suprachiasmatic nucleus (SCN) of the hypothalamus, alongside peripheral clocks found in various tissues, such as the liver, pancreas, and adipose tissue [53]. The central clock in the SCN is entrained by external cues, primarily light, and synchronizes the peripheral clocks through neural and hormonal signals. This hierarchical organization ensures that the entire body maintains a coordinated rhythmic pattern of metabolic processes [54]. The molecular basis of the circadian clock is characterized by an ensemble of fundamental clock genes, notably CLOCK, BMAL1, PER, and CRY, which establish transcriptional–translational feedback loops [55]. These genes and their protein products interact with each other to generate and maintain circadian oscillations with a period of approximately 24 h. The core clock genes not only regulate the circadian rhythm but also directly influence the expression of numerous metabolic genes involved in glucose and lipid metabolism [23] (Table 4). For example, CLOCK and BMAL1 have been shown to regulate the expression of glucose transporter 2 (GLUT2) and glucokinase, key enzymes involved in glucose uptake and utilization in the liver [23,56]. Additionally, these clock genes control the expression of sterol regulatory element-binding protein 1c (SREBP-1c), a transcription factor that regulates lipogenic genes and plays a central role in lipid metabolism [57]. The circadian clock system ensures the proper timing and coordination of metabolic processes, aligning them with daily feeding and activity cycles. Studies have shown that the expression of many metabolic genes, such as those involved in glucose and lipid metabolism, exhibit circadian rhythmicity [58]. For example, the expression of phosphoenolpyruvate carboxykinase (PEPCK), a key enzyme in gluconeogenesis, peaks during the inactive phase, whereas the expression of glucose-6-phosphatase (G6Pase), another essential gluconeogenic enzyme, peaks during the active phase [7,58,59]. This rhythmic expression of metabolic genes ensures that glucose production and utilization are optimized in accordance with energy demands of the body throughout the day. Similarly, the circadian clock system regulates lipid metabolism by controlling the expression of genes involved in lipogenesis, fatty acid oxidation, and cholesterol synthesis [57,60]. The expression of lipoprotein lipase (LPL), a key enzyme in the uptake and storage of triglycerides, exhibits circadian rhythmicity, with peak expression occurring during the active phase [7,57,61]. This rhythmic regulation of lipid metabolism ensures that energy storage and utilization are coordinated with daily feeding and activity patterns, thereby facilitating the integration of the metabolic process with the circadian rhythm. The disruption of the circadian clock system, whether due to genetic mutations or environmental factors such as sleep disruption or shift work, can result in metabolic dysregulation [62]. The development of metabolic disorders, including obesity, insulin resistance, and dyslipidemia, has been demonstrated in animal models with mutations in core clock genes. [7,59,60,62]. The circadian clock system plays a vital role in the regulation of metabolic processes, ensuring their proper timing and coordination with daily environmental cycles. The core clock genes regulate numerous metabolic genes, thereby maintaining the rhythmic expression of enzymes and transcription factors involved in glucose and lipid metabolism. The disruption of this complex system can lead to metabolic dysregulation and an increased risk of metabolic disorders. It is of paramount importance to gain a deeper understanding of the intricate interplay between the circadian clock and metabolism if we are to develop targeted interventions to prevent or treat metabolic diseases in the future.

### 5.2. Impact of Sleep Disruption on the Circadian Clock System

Sleep disruption, which has become increasingly prevalent in modern society due to factors such as shift work, jet lag, and the widespread use of electronic devices, can have a profound impact on the circadian clock system, leading to a wide range of adverse health consequences, particularly in relation to metabolic health [19]. It is therefore not surprising that sleep disruption can lead to a misalignment between the central and peripheral clocks, resulting in a wide range of adverse health effects. This misalignment can have serious consequences for metabolic health, as the peripheral clocks in organs such as the liver, pancreas, and adipose tissue play a crucial role in regulating glucose and lipid metabolism. Studies have shown that chronic circadian misalignment, such as that experienced by shift workers, is associated with an increased risk of metabolic disorders, including obesity, insulin resistance, and type 2 diabetes [19,44,63]. At the molecular level, the circadian clock is regulated by a set of core clock genes, including CLOCK, BMAL1, PER, and CRY [53,55]. These genes form transcriptional–translational feedback loops that generate and maintain circadian rhythms with a period of approximately 24 h. Sleep disruption has been shown to alter the expression of these core clock genes, leading to disruptions in the normal rhythmic patterns of physiological processes [11,58]. For instance, sleep deprivation has been found to decrease the expression of BMAL1 and increase the expression of PER genes in human peripheral blood mononuclear cells [64]. Such alterations in clock gene expression may have downstream effects on the regulation of metabolic genes, including those involved in glucose and lipid metabolism. In animal models, the disruption of clock genes has been associated with the development of metabolic disorders, including obesity and diabetes [65]. Melatonin, a hormone produced by the pineal gland, plays a crucial role in regulating the sleep–wake cycle and the circadian clock system. Melatonin secretion is suppressed by light exposure, particularly in the blue wavelength range, and typically peaks at night, promoting sleep. Sleep disruption, especially when associated with light exposure at night, can suppress melatonin secretion and disrupt the normal circadian rhythm of melatonin production [66]. Impaired melatonin secretion has been linked to several health problems, including sleep disorders, metabolic disorders, and cancer [66,67]. Melatonin has also been shown to have antioxidant and anti-inflammatory properties, and its disruption may contribute to the development of chronic diseases [66,67,68]. Sleep disruption can alter the timing of these metabolic processes, leading to impaired glucose and lipid metabolism. For example, studies have shown that the timing of food intake can affect the circadian rhythms of glucose tolerance and insulin sensitivity [66]. Individuals who consume their meals late at night, a common practice among shift workers and those with disrupted sleep patterns, have been found to exhibit impaired glucose tolerance and an elevated risk of developing T2DM [66,69,70]. The circadian clock system regulates the production and secretion of several hormones, including cortisol, growth hormone, and thyroid hormones [71]. The disruption of sleep patterns can alter the normal circadian rhythms of hormones, leading to the development of hormonal imbalances that can adversely affect metabolic health [5,9]. For instance, sleep deprivation has been demonstrated to increase cortisol levels, particularly dung the evening and early night, when cortisol levels typically decrease [9]. Elevated cortisol levels have been associated with insulin resistance, abdominal obesity, and other metabolic abnormalities [5,9].

### 5.3. Sleep Disruption and Glucose and Lipid Metabolism

Sleep disruption has been shown to have profound effects on glucose and lipid metabolism, contributing to the development of metabolic disorders such as obesity, insulin resistance, and type 2 diabetes. The molecular mechanisms underlying these effects are complex and multifaceted, involving alterations in gene expression, enzyme activity, and signaling pathways. Sleep disruption has been shown to alter the expression of numerous genes involved in glucose and lipid metabolism. These changes in gene expression are thought to be mediated, at least in part, by alterations in the activity of transcription factors and epigenetic modifications. One of the key transcription factors affected by sleep disruption is the circadian clock gene CLOCK. CLOCK, along with its partner BMAL1, regulates the expression of numerous genes involved in glucose and lipid metabolism, including those encoding enzymes involved in glucose and lipid synthesis and breakdown [65]. Sleep disruption has been shown to reduce the expression of CLOCK and BMAL1, leading to a decrease in the expression of their target genes [52,65] and has been shown to alter insulin signaling and glucose metabolism [65]. In a study by Möller-Levet et al. [64], a single week of insufficient sleep (5.7 h per night) led to changes in the expression of over 700 genes, many of which were involved in circadian rhythm and glucose metabolism. In addition to changes in gene expression, sleep disruption has also been shown to induce epigenetic modifications, such as DNA methylation and histone acetylation, which can further modulate gene expression [72]. These epigenetic changes may contribute to the persistent effects of sleep disruption on glucose and lipid metabolism, even after sleep patterns have normalized. There is accumulating evidence that sleep disruption alters the activity of several key enzymes involved in glucose and lipid metabolism, including the insulin signaling pathway and the AMP-activated protein kinase (AMPK) pathway [23,52,59,73]. Sleep disruption has been shown to impair insulin signaling by reducing the phosphorylation of key signaling molecules, such as insulin receptor substrate-1 (IRS-1) and Akt [11,62,74]. This impairment in insulin signaling can lead to insulin resistance and impaired glucose tolerance. The AMPK pathway is another important signaling pathway affected by sleep disruption [75]. AMPK is a cellular energy sensor that is activated in response to increased cellular energy demands, such as during exercise or fasting [76,77]. When activated, AMPK promotes glucose uptake, fatty acid oxidation, and mitochondrial biogenesis, while suppressing lipid synthesis and storage [76,78]. In a recent study, Hong et al. observed that mice subjected to sleep fragmentation (SF) had increased weight gain, impaired glucose regulation, inflammation, and decreased AMPK in white adipose tissue (WAT) [79]. This study showed that melatonin significantly improved these outcomes by mitigating SF-induced metabolic dysfunction, inflammation, and AMPK downregulation in adipose tissue. This suggests that the activation of AMPK may be a key mediator of the beneficial metabolic effects of melatonin following chronic SF. Mitochondria plays a critical role in glucose and lipid metabolism, serving as the primary site of glucose oxidation and fatty acid β-oxidation [80,81].

**Table 4:** The key molecular mechanisms of circadian clock system on the regulation of metabolic function.

Component	Function	Expression Pattern	Effect of Disruption	Tissue Specificity	Metabolic Impact	Therapeutic Implications	Reference
CLOCK /BMAL1	Core clock transcription factors; regulate metabolic genes	Peak expression during active phase	Altered amplitude and timing; disrupted rhythmicity	Hypothalamus, liver, adipose tissue, pancreas	Glucose dysregulation; lipid metabolism alterations	Chronotherapy potential; timing of interventions	[53,55]
PER/CRY	Negative feedback regulators; metabolic sensors	Peak during rest phase	Rhythm disruption; altered phase timing	Ubiquitous expression; tissue-specific patterns	Metabolic imbalance; energy homeostasis disruption	Timing of medication; meal timing importance	[55,64]
GLUT2/Glucokinase	Glucose transport; nutrient sensing	Diurnal rhythm in expression	Reduced expression; impaired function	Liver, pancreas β-cells, intestine	Impaired glucose uptake; altered insulin secretion	Glucose management strategies	[23,56]
SREBP-1c	Lipid homeostasis regulation; transcriptional control	Circadian oscillation	Dysregulation; phase shifts	Liver, adipose tissue	Altered lipid metabolism; fat storage changes	Lipid-lowering interventions	[57]
AMPK	Energy sensor; metabolic regulator	Activity follows feeding/fasting cycles	Reduced activation; timing disruption	Multiple tissues	Energy metabolism disruption; substrate utilization	Exercise timing; nutrient sensing	[75,76,77]

### 5.4. Sleep Disruption and Inflammation in Metabolic Dysfunction

Emerging research has revealed a complex interplay between sleep disturbances, chronic low-grade inflammation, and metabolic dysfunction [5,82,83]. This intricate relationship involves a myriad of molecular mechanisms linking circadian rhythms, inflammatory pathways, and metabolic regulation, painting a picture of interconnected biological processes with profound implication for human health (Table 5). One of the key molecular mechanisms linking sleep disruption to inflammation is the activation of the nuclear factor kappa B (NF-κB) pathway. NF-κB is a transcription factor that regulates the expression of numerous genes involved in the inflammatory response, including those encoding pro-inflammatory cytokines, chemokines, and adhesion molecules [25,84]. Sleep disruption has been shown to activate NF-κB signaling in various tissues, including peripheral blood mononuclear cells (PBMCs), adipose tissue, and the liver [18,25,26]. In a study by Irwin et al. [85,86], a single night of sleep loss led to a significant increase in NF-κB DNA-binding activity in PBMCs, which was accompanied by increased levels of pro-inflammatory cytokines, such as interleukin-6 (IL-6) and tumor necrosis factor-α (TNF-α). This upregulation of inflammatory markers was further exacerbated by enhanced sensitivity of Toll-like receptors (TLRs), particularly TLR4 [87,88]. TLRs are pattern recognition receptors that play a crucial role in the innate immune response [84]. Sleep loss increases the expression and sensitivity of TLR4, resulting in heightened inflammatory responses to both external pathogens (PAMPs) and internal damage signals (DAMPs) [88,89]. Recent investigations have implicated the NLRP3 (NOD-, LRR-, and pyrin domain-containing protein 3) inflammasome, a key component of the innate immune system, in sleep disruption-induced inflammation [90,91,92]. The NLRP3 inflammasome is a multiprotein complex that, upon activation, results in the production of the pro-inflammatory cytokines IL-1β and IL-18 [90,92]. Sleep disruption can prompt the assembly of the NLRP3 inflammasome complex, when it, in turn, leads to the production of these inflammatory mediators [25,92]. The activation of the NLRP3 inflammasome by sleep disruption likely involves multiple mechanisms [90,92,93]. Cellular stress induced by sleep loss, including mitochondrial dysfunction and increased reactive oxygen species (ROS) production, can serve as activation signals for NLRP3 [93]. Additionally, the circadian misalignment caused by sleep disruption may directly affect NLRP3 activation, as components of the inflammasome have been shown to be under circadian control [88,94]. Concurrently, sleep disruption has been demonstrated to disturb the delicate balance of appetite-regulating hormones. At the molecular level, sleep loss or sleep disruption has been linked to a reduction in leptin receptor sensitivity and an increase in ghrelin receptor expression, which together promote increased food intake and weight gain [95,96]. Leptin, produced by adipose tissue, exerts its effects on the hypothalamus, suppressing appetite and increasing energy expenditure. It seems that sleep loss may induce a state of leptin resistance, whereby despite adequate or even elevated leptin levels, the hormone’s appetite-suppressing effects are blunted [95]. This resistance may be mediated by the activation of endoplasmic reticulum stress, an increased expression of the suppressor of cytokine signaling 3 (SOCS3) and a reduced signal transducer and activator of transcription 3 (STAT3) phosphorylation [97]. In contrast, ghrelin has an orexinergic effect, with levels typically elevated before meals and suppressed after eating [98]. It has been demonstrated that sleep deprivation leads to an increase in ghrelin. This elevation of the ghrelin system may be a compensatory mechanism in response to the increased energy demands associated with extended wakefulness. However, it contributes to overeating and weight gain in the context of chronic sleep loss [99]. The inflammatory milieu created by sleep disruption is characterized by elevated levels of pro-inflammatory cytokines, which play a pivotal role in mediating the effects of sleep loss on metabolism [25,26,85,100]. Tumor necrosis factor-alpha (TNF-α) and interleukin-6 (IL-6) have been shown to interfere with insulin signaling through multiple mechanisms. These cytokines serve to activate inflammatory signaling cascades, including the c-Jun N-terminal kinases (JNK) and IKKβ pathways, which in turn lead to serine phosphorylation of IRS-1 [25,26,85]. Additionally, TNF-α and IL-6 have been demonstrated to induce the expression of SOCS3, which binds to the insulin receptor and IRS proteins, thereby inhibiting their function. SOCS3 has the capacity to target IRS proteins for proteasomal degradation, thereby exacerbating the impairment of insulin signaling [101]. This cytokine-induced insulin resistance creates a vicious cycle, as hyperglycemia resulting from impaired insulin action can further promote inflammation. Another cytokine, IL-1β, which is elevated in individuals who are sleep-deprived, impairs glucose-stimulated insulin secretion from pancreatic β-cells and induces insulin resistance in peripheral tissues. IL-1β has been demonstrated to activate the JNK and p38 mitogen-activated protein kinase (MAPK) pathways, leading to decreased expression of the glucose transporter GLUT4 and impaired insulin-stimulated glucose uptake [102]. Even C-reactive protein (CRP), which has traditionally been regarded as a marker of inflammation, may have a more direct role to play in metabolic dysfunction than was previously thought. Recent evidence suggests that CRP may bind to leptin, potentially impeding its ability to cross the blood–brain barrier and signal satiety, thus contributing to dysregulated appetite control [103]. Furthermore, CRP has been shown to induce insulin resistance in endothelial cells by increasing the phosphorylation of IRS-1 at inhibitory sites and reducing the activation of the insulin signaling mediator Akt [100,104], indicating that CRP may be a key contributor to the development of insulin resistance, rather than merely a passive marker of inflammation. In recent years, microRNAs (miRNAs) have emerged as important regulators in the complex interplay between sleep, inflammation, and metabolism [105,106]. This interconnected axis plays a crucial role in a multitude of physiological and pathological processes, particularly in conditions such as OSA and metabolic syndrome [107,108]. These small, non-coding RNAs regulate gene expression by binding to complementary sequences in target mRNAs, typically leading to mRNA degradation or translational repression [109]. Several microRNAs, including miR-16 and miR-146a, have been implicated in the regulation of both sleep and inflammatory processes [108]. For example, miR-16 has been shown to regulate the expression of the serotonin transporter, potentially influencing sleep–wake cycles [110]. The dysregulation of these miRNAs resulting from sleep disruption may contribute to altered inflammatory responses and metabolic dysfunction, thus adding another layer of complexity to this intricate biological tapestry. In conclusion, the molecular mechanisms underlying the interplay between sleep disruption, inflammation, and metabolic dysfunction constitute a complex and interconnected network. From alterations in circadian clock gene expression to the activation of inflammatory pathways, disruption of insulin signaling, and epigenetic modifications, sleep loss initiates a cascade of molecular events that collectively contribute to metabolic perturbations. As we continue to elucidate these intricate relationships, we gain not only a deeper understanding of the fundamental biology connecting sleep, inflammation, and metabolism but also valuable insights that may pave the way for novel therapeutic strategies.

## 6. Sleep Disruption and the Gut Microbiome in Metabolic Dysfunction

The complex interrelationship between sleep, the gut microbiome, and metabolic health has emerged as a compelling area of investigation in recent years, as evidenced by the literature [111,112] (Table 6). As our understanding of the human microbiome has expanded, it has become increasingly evident that sleep disturbances can significantly affect the composition and functionality of the gut microbiota, which may contribute to a range of metabolic disorders [111]. This intricate relationship represents a novel frontier in our comprehension of metabolic health and offers promising avenues for therapeutic interventions. It has been demonstrated that sleep loss and circadian misalignment induce rapid and significant alterations in the composition of the gut microbiome. A study conducted by Benedict et al. demonstrated that a mere two nights of partial sleep deprivation resulted in notable alterations in the composition of the gut microbiota of healthy young men [113]. In particular, an increase in the ratio of Firmicutes to Bacteroidetes was observed, a pattern that is often associated with obesity and metabolic disorders. This finding indicates that even brief disruptions to sleep patterns can exert discernible effects on the composition of the gut microbiota. The rapidity of these changes is particularly noteworthy, underscoring the dynamic nature of the gut microbiome and its sensitivity to environmental factors such as sleep. Further evidence can be found in animal studies, which allow for more controlled experimental conditions and longer-term observations. Poroyko et al. demonstrated that chronic sleep fragmentation in mice resulted in gut dysbiosis, characterized by an increase in pro-inflammatory bacteria and a decrease in beneficial bacteria [114]. This dysbiosis was accompanied by systemic and adipose tissue inflammation and insulin resistance, which underscores the potential metabolic consequences of sleep-induced alterations in the gut microbiome. Furthermore, the study revealed alterations in microbial metabolic pathways, particularly those involved in polysaccharide utilization and short-chain fatty acid production. These findings indicate that future interventions targeting the gut microbiota may prove an effective means of mitigating the adverse effects of chronic sleep disruption. The relationship between sleep and the gut microbiome is bidirectional and inextricably linked to circadian rhythms [111,112,115]. The composition and function of the gut microbiome exhibit diurnal oscillations, which can be disrupted by sleep disturbances and circadian misalignment [111,116]. A seminal study by Thaiss et al. demonstrated that circadian disruption induced by jet lag in both mice and humans resulted in dysbiosis and promoted glucose intolerance and obesity [117]. This study underscores the importance of maintaining regular sleep–wake cycles for the health and metabolism of the gut microbiome. It reveals a bidirectional feedback loop between host circadian rhythms and gut microbiome fluctuations, which can be disrupted by irregular sleep patterns. The mechanisms through which sleep disruption affect the gut microbiome and contribute to metabolic dysfunction are multifaceted and complex. One crucial pathway involves alterations in intestinal barrier function. It has been demonstrated that sleep deprivation is associated with an increase in intestinal permeability, which may contribute to the development of a condition known as “leaky gut” [111,117]. This increased permeability can result in the translocation of bacterial endotoxins, particularly lipopolysaccharides (LPS), into the bloodstream, thereby triggering systemic inflammation and contributing to insulin resistance and other metabolic disturbances. Additionally, the compromised intestinal barrier may permit the translocation of entire bacteria, thereby intensifying inflammatory responses and metabolic dysregulation. Sleep disturbances have also been shown to affect gut motility and transit time, which in turn influence nutrient absorption and bacterial colonization patterns [118,119]. Altered gut motility can result in alterations to the local gut environment, including changes in pH levels and oxygen availability [120]. This can result in the creation of an environment that is more conducive to the growth of certain bacterial species than others. Such alterations can have a cascading impact on microbial metabolite production and host–microbe interactions, which may contribute to the development of metabolic dysfunction [111,112]. Furthermore, sleep deprivation has been demonstrated to impair the secretion of hormones that play a pivotal role in regulating various physiological processes, including melatonin, cortisol, and growth hormone. These hormones regulate both the composition of the gut microbiota and metabolic processes [121,122]. Melatonin, for instance, has been shown to exert direct effects on the gut microbiome, influencing bacterial growth and biofilm formation [122,123]. The primary stress hormone, cortisol, has been demonstrated to impact gut permeability and immune function, thereby influencing the gut microbial environment, while growth hormone, which is primarily secreted during deep sleep, plays a pivotal role in metabolic processes and may exert an indirect influence on the gut microbiome through its effects on host physiology [124]. Such hormonal alterations can have significant implications for metabolic well-being, influencing a multitude of processes, including glucose regulation and lipid metabolism [123,124].

A further crucial factor to be taken into account is the influence of sleep deprivation on dietary patterns. A lack of sufficient sleep frequently results in alterations to eating behaviors, including an increased consumption of foods with high fat and sugar content [5]. Such dietary modifications can rapidly alter the composition of the gut microbiota, which may in turn exacerbate the metabolic consequences of sleep disruption. It has been demonstrated that high-fat diets can precipitate rapid alterations in the gut microbiome, promoting the proliferation of bacteria linked to obesity and inflammation [112,116,125]. The combination of sleep disruption and poor dietary choices may result in a state of metabolic dysfunction, whereby each factor serves to amplify the negative effects of the other. The sleep-induced alterations in the composition and function of the gut microbiome have been linked to various metabolic disturbances, including obesity, insulin resistance, and type 2 diabetes [111,112,116]. It has been demonstrated that sleep deprivation can induce anxiety-like behaviors and alter the composition of the gut microbiota, which may contribute to chronic inflammatory responses and influence the gut–brain axis [126]. Furthermore, a reduction in the duration of rapid eye movement (REM) sleep has been identified as an independent risk factor for an unfavorable glucose profile, with specific microbial taxa demonstrating correlations with both sleep duration and glucose levels [127]. The alterations in the gut microbiome resulting from sleep disturbances may contribute to the development of metabolic disturbances such as obesity, insulin resistance, and type 2 diabetes, underscoring the complex interrelationship between sleep, the gut microbiome, and metabolic health. A profound understanding of these intricate interrelationships may facilitate the development of innovative therapeutic interventions.

**Table 6:** The effect of sleep disruption on gut microbiome composition and associated metabolic alterations in human and animal studies.

Study Model	Population /Sample	Sleep Condition	Microbiome Changes	Metabolic Effects	Mechanisms	Reference
Human	Healthy young adults (*n* = 9)	2 nights partial sleep deprivation	↑Firmicutes/Bacteroidetes ratio; altered bacterial diversity	Impaired glucose metabolism; insulin sensitivity↓	Altered microbial metabolite production; changed gut permeability	[113]
Mouse	C57BL/6J mice	Chronic sleep fragmentation	↑Pro-inflammatory bacteria; ↓beneficial species; changed metabolic pathways	Insulin resistance; glucose intolerance; adipose tissue inflammation	Increased intestinal permeability; metabolic endotoxemia	[114]
Human/mouse	Humans and mice	Jet lag/circadian disruption	Dysbiosis; altered microbial rhythmicity	Glucose intolerance; weight gain; metabolic dysfunction	Circadian misalignment; disturbed feeding patterns	[117]
Mouse	Adult mice	Complete sleep deprivation	Altered diversity; changed bacterial composition	Anxiety-like behavior; metabolic changes	Gut–brain axis disruption; barrier dysfunction	[126]
Human	118 middle-aged volunteers	Reduced REM sleep	Christensenellaceae family positively associated with REM duration; Enterobacteriaceae family negatively associated with REM duration; iron metabolism	Glucose profile alterations	Sleep-microbiome interaction	[127]

Although the impact of sleep disruption on gut microbiota composition is evident, emerging evidence indicates that the gut microbiome also plays a crucial role in regulating sleep patterns. The gut microbiota has been shown to produce a variety of neuroactive compounds and metabolites that influence sleep architecture and quality through multiple pathways [128,129]. Recent research has elucidated several pivotal mechanisms: Firstly, gut bacteria are directly involved in the production of neurotransmitters such as GABA and serotonin, as well as the metabolism of tryptophan, a crucial precursor to melatonin production [129,130]. Secondly, microbiota-derived short-chain fatty acids (SCFAs) have been demonstrated to modulate sleep through the mediation of systemic inflammation and direct effects on the central nervous system via vagal nerve stimulation [128,131]. Thirdly, experimental studies have demonstrated that the depletion of microbiota leads to disrupted sleep patterns and reduced NREM sleep [132], while probiotic supplementation can improve sleep quality and increase the duration of slow-wave sleep [133]. The microbiome has been demonstrated to exert influence on circadian gene expression in a multitude of peripheral tissues, thereby establishing intricate feedback loops between bacterial metabolic activities and host circadian rhythms [134]. This bidirectional relationship suggests that disruptions in either sleep or gut microbiota can lead to perturbations in the other system, potentially creating a vicious cycle that exacerbates metabolic dysfunction.

As research in this field progresses, it is probable that a more detailed comprehension of the causal relationships between sleep, the gut microbiome, and metabolic health will be achieved [111]. It is recommended that future studies should concentrate on elucidating the precise mechanisms underlying these relationships and developing targeted interventions to interrupt the cycle of sleep disruption, gut dysbiosis, and metabolic dysfunction. This may entail longitudinal studies that monitor alterations in sleep patterns, gut microbiome composition, and metabolic markers over extended periods, in addition to interventional studies that assess the efficacy of diverse sleep improvement strategies on gut health and metabolic outcomes. In conclusion, the emerging field of research linking sleep disruption, alterations in the gut microbiome, and metabolic dysfunction represents a promising area for future investigation and the development of new therapeutic interventions.

## 7. Conclusions

**Figure 1:**
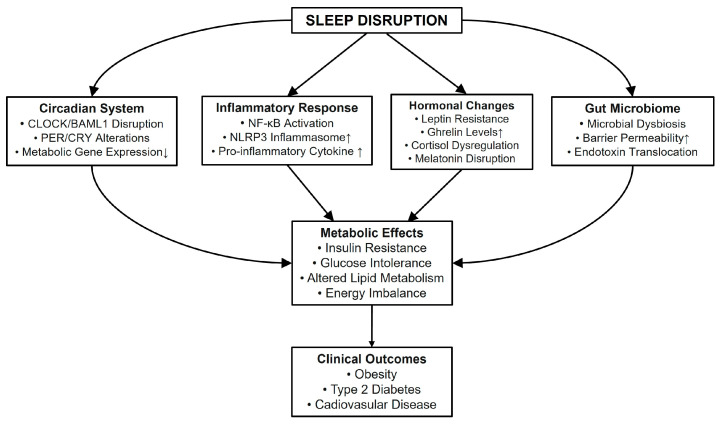
Molecular mechanisms linking sleep disruption to metabolic dysfunction and clinical outcomes.

Sleep disruption has been identified as a key factor contributing to metabolic dysfunction through a number of interconnected pathways (Figure 1). The evidence reviewed demonstrates that insufficient or poor-quality sleep impacts metabolic health through a number of mechanisms, including the disruption of circadian rhythms, alterations in glucose and lipid metabolism, the activation of inflammatory pathways, and the modulation of the gut microbiome. The molecular mechanisms underlying these relationships involve intricate interactions between core clock genes, metabolic regulators such as AMPK, pro-inflammatory mediators including NF-κB and the NLRP3 inflammasome, and alterations in the composition of the gut microbiota. A substantial body of clinical evidence has consistently demonstrated an association between sleep disruption and an increased risk of obesity, type 2 diabetes, and cardiovascular disease. The bidirectional relationship between sleep and metabolic health is further complicated by several additional factors, including diet, lifestyle, and environmental influences. An appreciation of these complex relationships has considerable implications for public health and clinical practice. It is recommended that future research should focus on the development of targeted interventions that address sleep disruption as a modifiable risk factor for metabolic disorders. Furthermore, further research is necessary to fully elucidate the causal pathways and identify potential therapeutic targets. It may be proposed that enhancing sleep quality and ensuring an adequate duration of sleep could prove an efficacious strategy for the prevention and treatment of metabolic disorders in modern society.

## Figures and Tables

**Table 1:** The relationship between sleep duration and obesity, as evidenced by epidemiological and meta-analytic studies.

Study Name	Population	Study Design	Follow-Up Duration	Key Findings	Effect Size/Risk Ratio	Reference
Nurses’ Health Study	60,000 female nurses aged 30–55; US-based	Prospective cohort	16 years	≤5 h sleep: +1.14 kg weight gain; each hour reduction: +0.35 kg gain; higher risk of obesity	0.35 kg per hour sleep reduction; linear relationship	[14]
Whitehall II	5021 British civil servants; mixed gender	Prospective cohort	5 years	Cross-sectional association but no prospective changes in BMI	No significant prospective effect	[3]
Bacaro et al.	154,936 participants	Meta-analysis	Various	≤6.5 h sleep increased obesity risk	OR = 1.41 (95% CI: 1.18–1.69)	[15]
Deng et al.	57,848 children/adolescents	Meta-analysis	Various	Short sleep increased obesity risk	RR = 1.57 (95% CI: 1.36–1.81)	[16]

**Table 5:** The key molecular pathways linked to sleep disruption and inflammation in metabolic dysfunction.

Pathway	Function	Metabolic Consequences	Therapeutic Targets	Biomarkers	Reference
NF-κB signaling	Inflammatory response regulation and immune homeostasis	Insulin resistance development; enhanced adipose inflammation; Increased hepatic lipogenesis	IKK inhibitors; natural compounds; anti-inflammatory agents	p-NF-κB; nuclear p65; IκB levels	[25,84]
NLRP3 inflammasome	Innate immune response and metabolic sensing	Impaired insulin signaling; pancreatic β-cell dysfunction; enhanced adipose inflammation	NLRP3 inhibitors; IL-1β antagonists; ROS scavengers	IL-1β levels; IL-18 levels; ASC specks	[90,91,92]
TLR4 signaling	Pattern recognition and innate immunity modulation	Systemic insulin resistance; altered lipid metabolism; disrupted glucose homeostasis	TLR4 antagonists; pathway inhibitors; metabolic targets	TLR4 expression; inflammatory markers	[87,88]
Pro-inflammatory cytokines	Immune signaling and metabolic regulation	Impaired insulin signaling; reduced glucose uptake; altered metabolism	Cytokine inhibitors; receptor antagonists; anti-inflammatory agents	TNF-α; IL-6; IL-1β	[25,26,85]
CRP pathway	Acute phase protein production and inflammatory response	Leptin resistance; reduced insulin sensitivity	Anti-inflammatory agents; metabolic modulators	hs-CRP; inflammatory panels	[103,104]

## Data Availability

All data supporting the results are presented in the manuscript. Any other inquiries can be directed to the corresponding authors via email.
